# Organic–inorganic nanocrystal reductase to promote green asymmetric synthesis†

**DOI:** 10.1039/d0ra03160g

**Published:** 2020-08-20

**Authors:** Kotchakorn T.sriwong, Afifa Ayu Koesoema, Tomoko Matsuda

**Affiliations:** Department of Life Science and Technology, School of Life Science and Technology, Tokyo Institute of Technology 4259 Nagatsuta-cho, Midori-ku Yokohama 226-8501 Japan tmatsuda@bio.titech.ac.jp +81-45-924-5757 +81-45-924-5757

## Abstract

An acetophenone reductase from *Geotrichum candidum* (*Gc*APRD) was immobilized by the organic–inorganic nanocrystal method. The *Gc*APRD nanocrystal presented improved stability and recyclability compared with those of the free *Gc*APRD. Moreover, the *Gc*APRD nanocrystal reduced broad kinds of ketones with excellent enantioselectivities to produce beneficial chiral alcohols such as (*S*)-1-(3′,4′-dichlorophenyl)ethanol with >99% yield and >99% ee. The robust and versatile properties of the *Gc*APRD nanocrystal demonstrated an approach to promote green asymmetric synthesis and sustainable chemistry.

## Abstract 2

*Geotrichum candidum* acetophenone reductase (*Gc*APRD) nanocrystal reduces broad kinds of ketones to their corresponding (*S*)-alcohols with excellent enantioselectivity.

## Graphical abstract

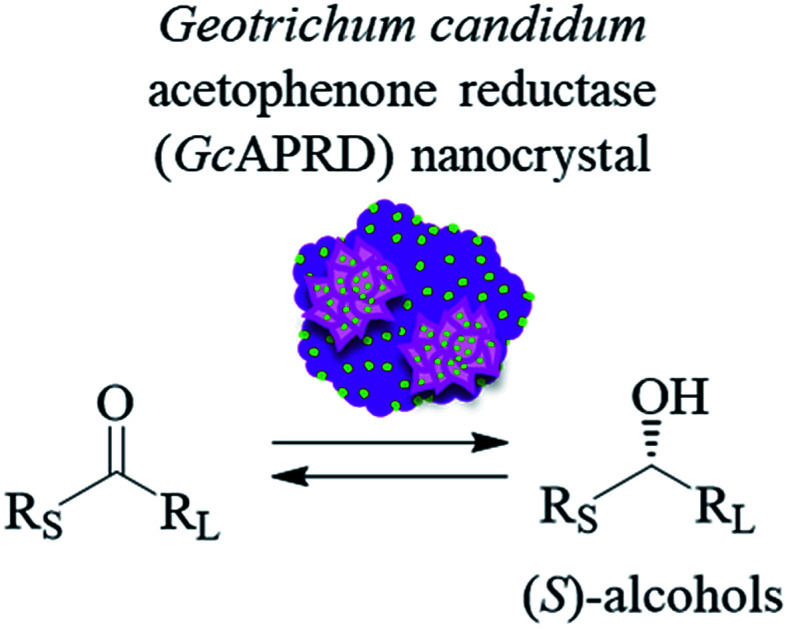

## 1. Introduction

Biocatalysts have presented superior properties for green and sustainable synthesis with their high selectivity, turnover rate, and efficiency.^1,2^ For example, an alcohol dehydrogenase (ADH) from *Rhodococcus ruber* DSM 44541 expressed in *Arxula adeninivorans* is able to enantioselectively reduce *p*-chloroacetophenone with a remarkable turnover rate (*K*_cat_) of 432 s^−1^.^3,4^ Thus, the biocatalysts have played an essential role in an extensive area of industries such as fine and bulk chemical, pharmaceutical, food and beverage, cosmetic, textile, and pulp and paper.^5^ Meanwhile, the needs for chiral compounds have risen continuously, especially in the pharmaceutical fields, due to the different biological activities and toxicities that may be exhibited by each enantiomer of the chiral compounds.^6,7^

Asymmetric reduction of ketones by chemical and biological catalysts has been considered as a promising method to produce chiral alcohols.^8–10^ Enzymes catalyzing asymmetric reduction reactions have been widely studied due to their high specificity property, and the well-known enzyme used for this purpose is ADH.^10^ An acetophenone reductase from *Geotrichum candidum* NBRC 4597 (*Gc*APRD) is a novel ADH that reduces ketones to their corresponding (*S*)-alcohols.^11^ The *Gc*APRD has versatile and robust properties such as excellent enantioselectivity, broad substrate specificity, and non-aqueous solvents tolerance that make it potential for industrial applications.^12–18^ However, the free form of *Gc*APRD has limitations to recycle and operate in a flow process.

Enzyme immobilization is a method that improves stability and recyclability of the enzyme allowing the enzyme to operate under wide conditions.^2^ In most cases, the enzyme is confined with the immobilization supports that limits their mobility.^19^ Various techniques have been addressed to immobilize the enzyme, including the organic–inorganic nanocrystal method, first reported by Ge *et al.* in 2012.^20–22^ This method has been proven to be effective in immobilizing various biomolecules such as proteins, peptides, and antibodies.^21,22^ Moreover, limitations faced by other immobilization methods, such as insufficient binding of protein on support and severe loss of activities, have been overcome by the organic–inorganic nanocrystal method.^19,23^ Thereby, several studies have applied this method to immobilize broader kinds of biomolecules.^21^ Enzymes have been one of the most widespread biomolecules immobilized by this method for examples α-lactalbumin,^22^ laccase,^22^ carbonic anhydrase,^22^ lipases,^22,24–26^ α-amylases,^27^ papain,^28,29^ and α-acetolactate decarboxylases.^30^ However, only a few studies have focused on the immobilization of alcohol dehydrogenase (ADH) by this method,^31^ particularly for asymmetric synthesis,^32^ and there is no example yet of versatile ADH nanocrystals with broad substrate specificities.

Thus, this research aimed to immobilize the *Gc*APRD by using the organic–inorganic nanocrystal method. The preparation conditions of *Gc*APRD nanocrystal were optimized, and the properties of the *Gc*APRD nanocrystal were characterized. The *Gc*APRD nanocrystal showed improved stability and recyclability while retaining excellent enantioselectivity. To the best of our knowledge, this is the first study to report an ADH nanocrystal with broad substrate specificity.

## 2. Materials and methods

### 2.1 Reagents and materials

*Gc*APRD wild type was prepared according to the previous procedure.^12^ Chemicals for cell cultivation, enzyme purification, and preparation of the *Gc*APRD nanocrystal were supplied from Nacalai Tesque (Japan), except for 4-(2-hydroxyethyl)-1-piperazine ethanesulfonic acid (HEPES) and dithiothreitol (DTT), which were purchased from Sigma Aldrich (USA) and Wako (Japan), respectively. Protein concentration measurement reagent was supplied from Bio-Rad (USA). Commercial grade solvents, ketones, and alcohol standards (1b–3b) were purchased from Nacalai Tesque (Japan). Previously prepared alcohol (4b–7b) and chiral ester (2b–4b and 7b) standards were used in this study.^33–35^

### 2.2 Apparatus

Ultraviolet-visible spectroscopic analysis was conducted in a UV-1900-UV-Visible spectrophotometer from Shimadzu (Japan). Chiral gas chromatography (GC) was performed on a GC-14B equipped with flame ionization detector and a CP-Chirasil-Dex-CB column (Varian 0.32 mm × 0.25 μm × 50 m) using He carrier gas (5 mL min^−1^, head pressure: 274 kPa, injector: 180 °C, detector: 180 °C) (Shimadzu, Japan). The morphology and energy dispersive X-ray spectroscopy (EDX) analysis were studied by Bench-top Scanning Electron Microscope proX supplied by Phenom-World (Netherlands). Thermo Gravimetric Analysis (TGA) was performed on TG-DTA, DTG-60 (Shimadzu, Japan). The optical rotation value was measured by JASCO P-2200 Polarimeter with a 10 cm path-length cell. The ^1^H-NMR analysis was performed on a Bruker Biospin AVANCE III 400 spectrometer at 400 MHz in CDCl_3_.

### 2.3 *Gc*APRD nanocrystal preparation

The phosphate-buffered saline (PBS) containing *Gc*APRD was mixed with a metal ion by pipetting for 5 times gently, incubated at 4 °C for 24 h, and centrifuged at 4 °C, 12 000 rpm for 5 min. The detailed experimental conditions are shown in the ESI (Table S1†). The supernatant was removed to measure the residual protein amount by Bradford method,^36^ and to calculate the immobilization yield (1). The precipitant was washed by distilled water and resuspended in PBS (*Gc*APRD nanocrystal).1
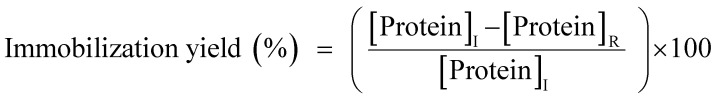
[Protein]_I_ = initial protein concentration (mg mL^−1^), [Protein]_R_ = concentration of residual protein in the supernatant after the *Gc*APRD nanocrystal formation and centrifugation (mg mL^−1^).

### 2.4 Relative reaction yield and activity measurement of enzyme

The relative reaction yield measurement to investigate the optimum immobilization conditions was conducted by mixing acetophenone 1a (5.0 mM), 2-propanol (15% v/v), free *Gc*APRD (8.30–16.50 μg of protein per mL) or *Gc*APRD nanocrystal (8.30–33.0 μg of protein per mL), NAD^+^ (0.20 mM), and HEPES–NaOH buffer (0.10 M, pH 7.2) up to 3 mL. For the investigation of the optimum immobilization conditions, the reaction was performed at 37 °C, 200 rpm for 10 min. The product formed (1b) was extracted with diethyl ether and analyzed by GC, using 3-methyl-1-butanol as an internal standard.

Activity measurement to investigate the temperature and pH profile, and the stability of *Gc*APRD nanocrystal were assayed by using UV-spectrophotometer. All assays were performed at 37 °C in 1.0 mL HEPES–NaOH buffer (0.10 M, pH 7.2), consisting of 1a (5.0 mM) except for the pH profile experiment (5.6 mM), NADH (0.28 mM), and *Gc*APRD nanocrystal (0.80–4.00 μg of protein per mL). Initial velocity was determined by detecting the decrease of NADH at 340 nm for 132 s.

The recyclability was assessed by batch process. The reaction mixture consisted of 1a (3.0 mM), 2-propanol (15% v/v), *Gc*APRD nanocrystal (630 μg of protein), NAD^+^ (0.20 mM), and HEPES–NaOH buffer (0.10 M, pH 7.2) up to 1.5 mL. The reaction in each cycle was conducted at 30 °C, 200 rpm for up to 180 min. Afterward, the reaction mixture was centrifuged at 4 °C, 16 000 rpm for 5 min. The supernatant was removed, extracted with diethyl ether, and analyzed by GC. The precipitant was washed by the reaction mixture with the absence of NAD^+^, and proceeded the next reaction cycle.

### 2.5 Micromole scale asymmetric reduction of ketones by *Gc*APRD nanocrystal

The experiment was conducted based on the previously reported procedure.^13^ Micromole scale reductions were performed in a 3.0 mL reaction mixture, which consists of 1a–7a (5.0 mM), 2-propanol (15% v/v), NAD^+^ (5.0 mM), HEPES–NaOH buffer (0.10 M, pH 7.2), and *Gc*APRD nanocrystal with the amount needed to achieve 1.0 μmol min^−1^ mL^−1^ reduction of each substrate to its corresponding alcohol for 1a–6a, and 350 μg of protein/mL for 7a. The mixture was incubated at 30 °C, 200 rpm for 3 h. A portion of the mixture was extracted with diethyl ether to determine the reaction yield by GC analysis. The enantioselectivity excess (ee) values and the absolute configurations of 1b, 5b, and 6b were determined by comparing their GC retention times with the corresponding authentic samples prepared before.^35^ Meanwhile, for 2b–4b and 7b, the reaction mixtures were extracted by dichloromethane, dried over MgSO_4_, and used for propionylation by propionyl chloride reaction. ee values and absolute configurations were determined by comparing the GC retention times of propionates of 2b–4b and 7b with the corresponding authentic samples prepared before.^33,34^ The chiral GC retention time of ketone, alcohol, and ester standards are given in Table S2.†

### 2.6 Preparative scale asymmetric reduction of a ketone by *Gc*APRD nanocrystal

The reaction was performed in a 100 mL scale consisting of 6a (197 mg, 10.0 mM), 2-propanol (3.0% v/v), NAD^+^ (1.4 mM), HEPES–NaOH buffer (0.10 M, pH 7.2), and *Gc*APRD nanocrystal (2 mg of protein). The mixture was incubated at 30 °C, 130 rpm for 18 h, and extracted by diethyl ether for 3 times, dried over MgSO_4_, filtered, and evaporated. The corresponding alcohol was purified by silica gel column chromatography (hexane : ethyl acetate, 3 : 1) to afford (*S*)-6b (165 mg, 0.87 mmol, 84%), and characterized by ^1^H-NMR analysis. The ^1^H-NMR spectra of alcohol (*S*)-6b was in agreement with that reported in the literature.^37^ ee was determined by chiral GC analysis, and the absolute configuration was determined by comparing the optical rotation value of product with the literature.^38^ [*α*]^20^_D_ = −38.19 (*c* = 1.04, CHCl_3_, ee = >99%); lit^38^ (*S*)-6b: [*α*]^20^_D_ = −33.70 (*c* = 1.44, CHCl_3_, ee = 91%). ^1^H-NMR (400 MHz, CDCl_3_, 25 °C, TMS): *δ* = 7.48 (d, *J* = 2.0 Hz, 1H, CH), 7.41 (d, *J* = 8.3 Hz, 1H, CH), 7.20 (dd, *J* = 8.2 Hz, 2.0 Hz, 1H, CH), 4.84–4.90 (m, 1H, CH), 1.82–1.83 (m, 1H, OH), 1.48 (d, *J* = 6.48 Hz, 3H, CH_3_).

### 2.7 *Gc*APRD nanocrystal characterization by scanning electron microscopy (SEM) and EDX analysis, and TGA

The prepared *Gc*APRD nanocrystal was washed by distilled water several times to remove the residual phosphate and metal ion prior to drying at room temperature. The dried *Gc*APRD nanocrystal was used for SEM and EDX analysis, and TGA.

## 3. Results and discussions

### 3.1 Optimization of *Gc*APRD nanocrystal synthesis

**Fig. 1:**
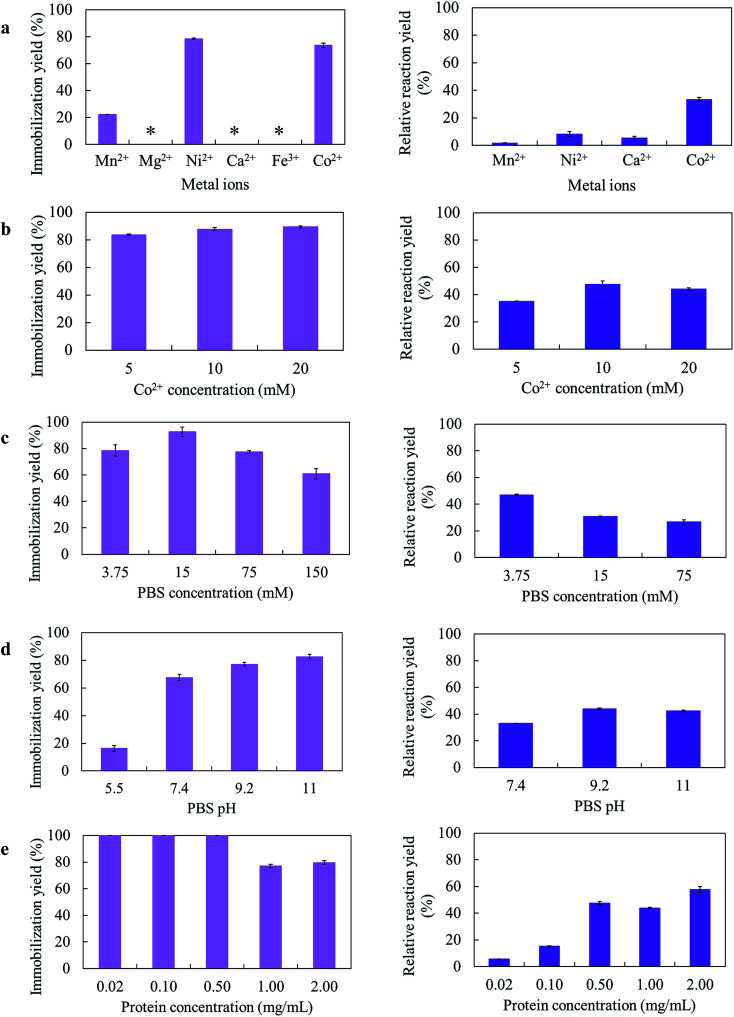
Optimization of *Gc*APRD nanocrystal preparation (a) metal ions, (b) metal ion (Co^2+^) concentration, (c) PBS concentration, (d) PBS pH, and (e) protein concentration. Left and right column depict the immobilization yields and relative reaction yields, respectively. The reaction yield of free *Gc*APRD was set to be 100%. *Very small precipitation was found. The preparation and reaction conditions are described in the section 2.4.

Several parameters for the *Gc*APRD nanocrystal preparation, such as kinds of metal ions, metal concentration, PBS concentration, PBS pH, and protein concentration, were varied. The immobilization yields and relative reaction yields are shown in Fig. 1.

Different amino acid residues in each biomolecule present different affinities with various metal ions, and the appropriate metal ion concentration is necessary for the nucleation.^21^ In this research, 6 metal ions were used to prepare the *Gc*APRD nanocrystal (Fig. 1a). It was found that the immobilization yield was high when using Co^2+^ or Ni^2+^ as a metal ion. Nonetheless, only the *Gc*APRD nanocrystal synthesized by Co^2+^ retained sufficient activity. It was assumed that the His-tag located in the C-terminus of the *Gc*APRD acted as a binder with Co^2+^ or Ni^2+^ ion, which was comparable to the discovery of López-Gallego and Yate's.^31^ They reported that the presence of His-tagged protein is mandatory as the driving force for the crystal formation. The His-tag binds Co^2+^ ions *via* the coordination bond and forms a nucleation site, which initiates the crystal formation.^31^ Although Ni^2+^ could form the crystal with His-tagged protein, high concentration of nickel could cause the enzyme deactivation (Fig. S1†). To investigate the importance of Co^2+^ and His-tagged *Gc*APRD bonding, the formed *Gc*APRD nanocrystal was suspended in 500 mM imidazole in 5 mM PBS to break the Co^2+^ and His-tag bond, thus eluting the protein. After the elution, the *Gc*APRD nanocrystal was apparently detached, suggested that Co^2+^ and His-tag bonding played an important role in the *Gc*APRD nanocrystal formation (Fig. S2†). Additionally, the metal concentration was optimized to 10 mM (Fig. 1b), comparable with Kim *et al.*’s study (10 mM of cobalt for the formation of BSA–inorganic hybrid nanoflower).^39^

PBS concentration is another important parameter for the nanocrystal formation.^28^ Thereby, we investigated the effect of PBS concentration on the *Gc*APRD nanocrystal formation (Fig. 1c). The prepared *Gc*APRD nanocrystal performed the highest relative reaction yield at 3.75 mM PBS. However, the optimum PBS concentration is different depending on the biomolecule and other synthesis parameters.^40,41^ Synthesis pH is an essential parameter influencing the nanocrystal formation.^29,41,42^ Thus, this study investigated the effect of PBS pH on the *Gc*APRD nanocrystal formation (Fig. 1d). The immobilization yield of *Gc*APRD was low in acidic conditions (pH 5.5), and higher in higher pH (pH 7.4, 9.2, and 11.0). It was proposed that in acidic conditions, the biomolecule is highly protonated, causing strong repulsions with the positive charges of the transition metal (*i.e.*, Cu^2+^and Co^2+^).^43^ Joshua *et al.*, reported that protonating the imidazole nitrogen atom of the histidine residue (pKa 6.0) by lowering the pH to 6.0 can disrupt the coordination bond between histidine and Co^2+^.^44^ Altinkaynak *et al.* also reported an easier synthesis of lactoperoxidase hybrid nanoflower at pH 9.0 and 10.0.^19^ The *Gc*APRD nanocrystal showed the best relative reaction yield in pH 9.2, and pH 11.0.

The presence of biomolecule is mandatory for the hybrid nanocrystal formation.^21,22^ In this study, the protein concentration in the *Gc*APRD nanocrystal formation was varied to 0.02, 0.10, 0.05, 1.00, and 2.00 mg mL^−1^ (Fig. 1e). The experiments demonstrated that at the protein concentration of 0.05 mg mL^−1^ and below, the enzyme was entirely immobilized (immobilization yield >99%). On the other hand, the protein concentration of more than 1.00 mg mL^−1^ resulted in a decrease in immobilization yield, but protein immobilized per metal amount was high. Nevertheless, the relative reaction yield result showed that the reaction proceeded better when the synthesized protein concentrations were more than 0.50 mg mL^−1^, and the greatest relative reaction yield was found on the 2.00 mg mL^−1^ of protein. Ge *et al.* reported that different concentration of protein has a different nanocrystal formation mechanism. The lower concentration of protein causes fewer nucleation sites, leading to a larger nanocrystal with a more complex structure.^22^ At last, it was confirmed that the active *Gc*APRD was mandatory to the *Gc*APRD nanocrystal activity (ESI, section 4†). To understand this phenomenon clearly, SEM analysis on *Gc*APRD nanocrystal synthesized with various protein concentrations were performed as described in section 3.2.

### 3.2 Morphology analysis by SEM

**Fig. 2:**
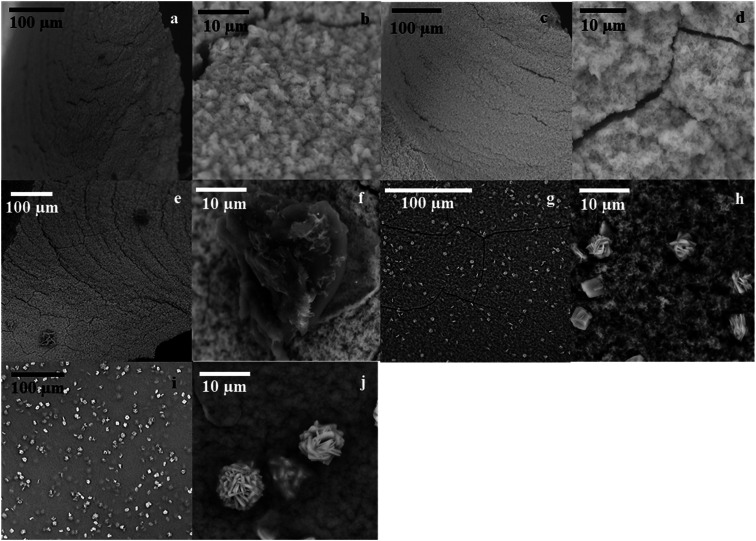
SEM images of *Gc*APRD nanocrystal formed under various protein concentrations, (a and b) control (no enzyme), (c and d) 0.02 mg mL^−1^, (e and f) 0.10 mg mL^−1^, (g and h) 0.50 mg mL^−1^, and (i and j) 2.00 mg mL^−1^.

**Fig. 3:**
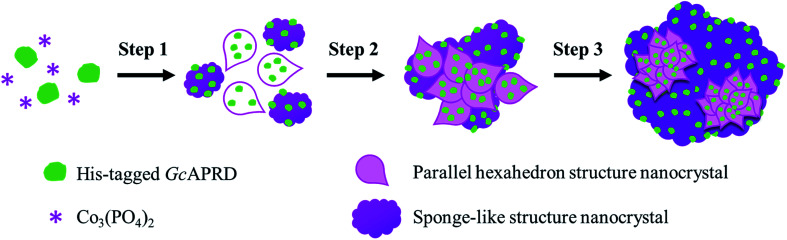
The proposed formation mechanism of *Gc*APRD nanocrystal, comprised of 3 steps: (1) nucleation, (2) aggregation, and (3) anisotropic growth.

SEM analysis on *Gc*APRD nanocrystal synthesized with various protein concentrations was performed to understand its effect in the immobilization yield and relative reaction yield. As shown in Fig. 2, it was found that without enzyme, the cobalt-phosphate crystal was formed (Fig. 2a and b). In the presence of 0.02 mg mL^−1^ (Fig. 2c and d) and 0.10 mg mL^−1^ (Fig. 2e and f) of protein, the surfaces of the crystals were similar to the control, assuming that the small amount of protein was buried underneath the cobalt–phosphate crystals. Therefore, the mass transfer between the hindered enzyme and the substrate was limited, and low relative reaction yields were observed for the nanocrystals synthesized with 0.02 and 0.10 mg mL^−1^ of protein. The surfaces of *Gc*APRD nanocrystals synthesized with 0.50 mg mL^−1^ (Fig. 2g–h) and 2.00 mg mL^−1^ (Fig. 2i–j) protein were different from the control and displaying a parallel hexahedron structure assembled to a flower-like structure mixed with sponge-like structure. The proposed formation mechanism of the *Gc*APRD nanocrystal synthesized with 0.50 and 2.00 mg mL^−1^ of protein is presented in Fig. 3. There may be 3 steps, comprised of nucleation, aggregation, and anisotropic growth. A similar structure was reported previously by Wang *et al.* in the formation of nanocrystals with the presence of 2.00 mg mL^−1^ protein.^27,31^ The high relative reaction yield suggested that the *Gc*APRD was gathered on the surface of the nanostructure, and the enzymes were not buried. In addition, the *Gc*APRD nanocrystal was characterized by EDX analysis and TGA to examine the chemical composition and structure of the *Gc*APRD nanocrystal as in Fig. S3–S5 (ESI,† section 5).

### 3.3 Temperature and pH profile, and stability of *Gc*APRD nanocrystal

**Fig. 4:**
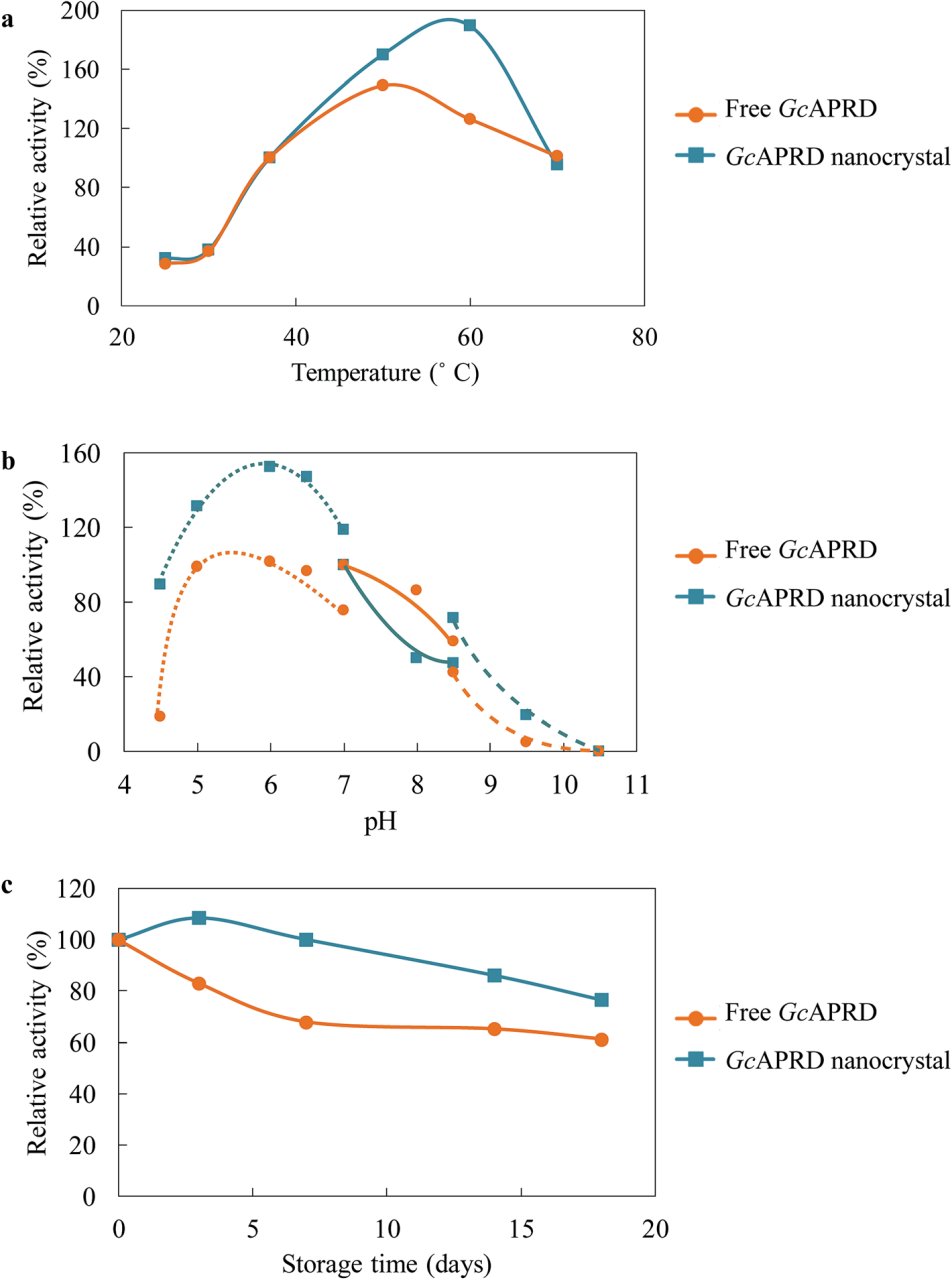
Temperature and pH profile, and stability of free *Gc*APRD (orange-circle line) and *Gc*APRD nanocrystal (blue-square line). (a) temperature profile, (b) pH profile, and (c) storage stability. Enzyme activities of free *Gc*APRD and *Gc*APRD nanocrystal at 37 °C for (a), HEPES–NaOH buffer (pH 7.0) for (b), and day 0 for (c) were set to be 100%. For the pH profile, the enzyme activity was measured in MES–NaOH buffer (0.10 M) (dotted line), HEPES–NaOH buffer (0.10 M) (line), and glycine–NaOH buffer (0.10 M) (broken line). The activity assay is described in the section 2.4.

Immobilization has been reported to improve the temperature and pH profile, as well as the stability of the enzyme.^2,31,40,41^ The temperature profile of *Gc*APRD nanocrystal and free *Gc*APRD was examined (Fig. 4a). Both free *Gc*APRD and *Gc*APRD nanocrystal demonstrated poor activity at low temperatures (20–30 °C). The activities of both enzymes were increased in higher temperatures. The free *Gc*APRD performed the highest relative activity at 50 °C (170%), and the activity gradually decreased at the temperature above 60 °C. To our delight, the *Gc*APRD nanocrystal presented the improved temperature profile as the highest relative activity was observed at 60 °C (189%). At the same time, the effect of pH on the *Gc*APRD nanocrystal activity was investigated, and it showed less sensitivity than the free *Gc*APRD in acidic conditions (Fig. 4b). The relative activity of the free *Gc*APRD dramatically decreased from 99% to 18% when the pH was lowered from 5.0 to 4.5, while the *Gc*APRD nanocrystal's relative activity moderately decreased from 132% to 89%. The storage stability of the enzymes at 4 °C was investigated. As shown in Fig. 4c, after 7 days, the residual activity of the free *Gc*APRD was 68%, while the *Gc*APRD nanocrystal still retained 100% activity. Also, after 18 days, the free *Gc*APRD's residual activity was 61%, while the *Gc*APRD nanocrystal had 76% of residual activity.

### 3.4 Recyclability of *Gc*APRD nanocrystal

**Fig. 5:**
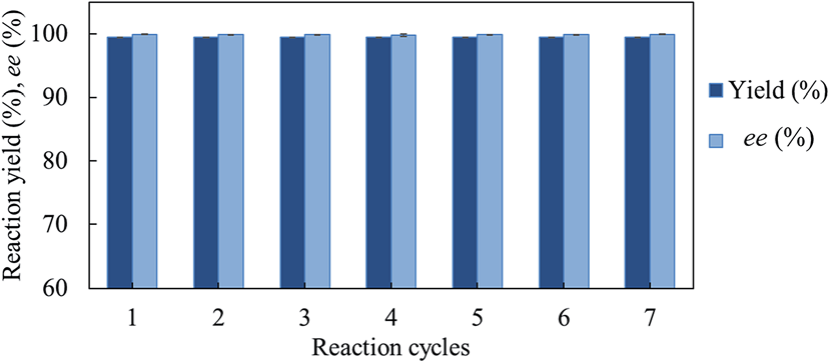
Recyclability of *Gc*APRD nanocrystal. The reaction condition is described in the section 2.4.

We investigated the recyclability of the *Gc*APRD nanocrystal by performing the reduction of 1a in batch process. The *Gc*APRD nanocrystal was able to be recycled with noticeable high reduction yield (>99%) and excellent enantioselectivity (>99%) up to 7 reaction cycles (Fig. 5). This result proved the superior recyclable properties of the *Gc*APRD nanocrystal, which is a promising approach to further applications.

### 3.5 Substrate specificity of *Gc*APRD nanocrystal

Excellent enantioselectivity and broad substrate specificity are one of the most crucial aspects of the enzyme utilization for industrial processes.^10^ The *Gc*APRD nanocrystal was tested to reduce broad kinds of ketones (Table 1). 1a, as a model substrate for the *Gc*APRD reduction, was reduced by *Gc*APRD nanocrystal with >99% yield and >99% ee (*S*). The *Gc*APRD nanocrystal also reduced challenging aliphatic ketones 2a–4a with high yields and enantioselectivities (up to >99% ee (*S*)). Moreover, the *Gc*APRD nanocrystal was able to enantioselectively reduce “difficult to resolve” ketone 3a with only one carbon difference between side chains adjacent to the carbonyl carbon. The enantioselectivities reported for the free enzyme were retained perfectly.^9,12,13^

**Table 1:** Reduction of ketones by *Gc*APRD nanocrystal^a^

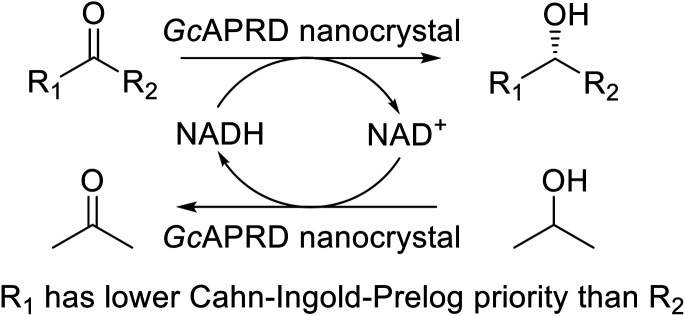		
Substrate	Yield (%)	ee (%)
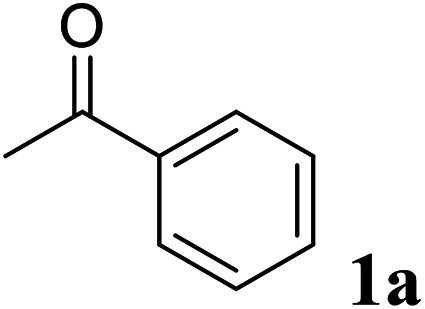	>99	>99 (*S*)
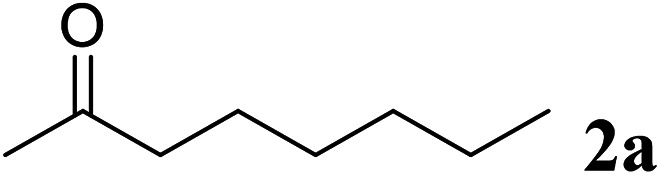	>99	>99 (*S*)
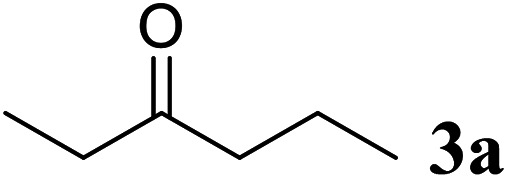	74	99 (*S*)
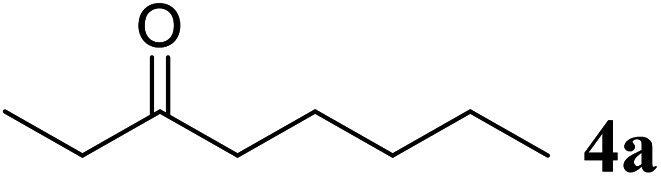	77	>99 (*S*)
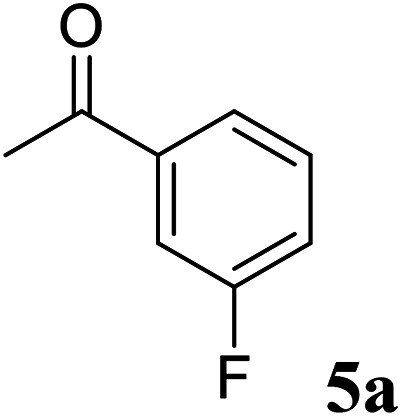	>99	>99 (*S*)
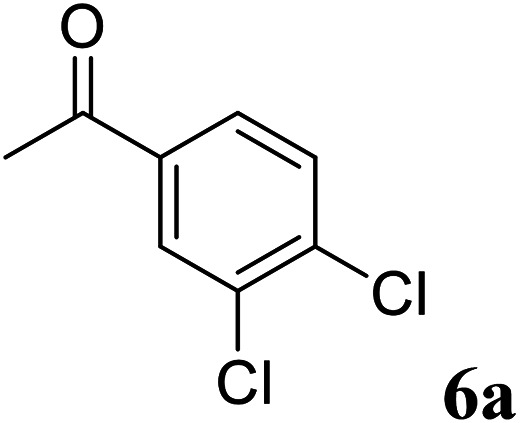	>99	>99 (*S*)
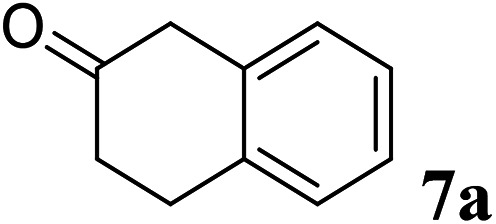	91	96 (*S*)

a The reaction conditions are described in the section 2.5.

The *Gc*APRD nanocrystal produced beneficial chiral alcohols with excellent enantioselectivities (ee up to >99% (*S*)) such as (*S*)-5b (intermediate of EGFR and HER2 kinase inhibitors, a potential cancer treatment agent),^45^ (*S*)-6b (intermediate of sertraline, an obsessive-compulsive disorder medication),^46^ and (*S*)-7b (intermediate of aminotetralin, which can be used to stimulate dopamine, serotonin, and melatonin receptors).^47,48^ We were also able to isolate (*S*)-6b with 84% isolated yield, in a preparative scale reaction catalyzed by the *Gc*APRD nanocrystal. To the best of our knowledge, this is the first study to report an ADH nanocrystal with broad substrate specificity.

## 4. Conclusions

We successfully immobilized a novel ADH, *Gc*APRD, by the organic–inorganic nanocrystal method. The *Gc*APRD nanocrystal presented improved temperature and pH profile, and stability compared with those of the free *Gc*APRD. The *Gc*APRD nanocrystal overcame the recyclability limitation of the free *Gc*APRD. It could be recycled up to 7 cycles with remarkably high yield and excellent enantioselectivity. The *Gc*APRD nanocrystal reduced broad kinds of ketones to produce valuable chiral alcohols. The robust and versatile properties of the *Gc*APRD nanocrystal made it a promising and sustainable catalyst for industrial applications. Future studies will investigate the reaction of *Gc*APRD nanocrystal in non-aqueous solvent and perform cascade reactions to allow the production of broader kinds of beneficial compounds.

## Conflicts of interest

There are no conflicts to declare.

## Supplementary Material

RA-010-D0RA03160G-s001
