# Supplementary material for: Organic–inorganic nanocrystal reductase to promote green asymmetric synthesis
Source: RSC Adv. 2020 Aug 20;10(51):30953–60. doi: 10.1039/d0ra03160g (PMC9056328; doi:10.1039/d0ra03160g)
Supplement: RA-010-D0RA03160G-s001 [file RA-010-D0RA03160G-s001.pdf]

Electronic Supplementary Material (ESI) for RSC Advances.
This journal is © The Royal Society of Chemistry 2020

Supporting Information

Organic-inorganic nanocrystal reductase to promote green asymmetric synthesis

Kotchakorn T.sriwong, Afifa Ayu Koesoema, Tomoko Matsuda

Department of Life Science and Technology, School of Life Science and Technology, Tokyo Institute of Technology, 4259 Nagatsuta-cho, Midori-ku, Yokohama, 226-8501 Japan

List of contents

1. Experimental section 2

Table S1 Conditions of *Gc*APRD nanocrystal preparation....... 2

Table S2 Chiral GC retention time of ketone, alcohol, and ester standards 2

2. Relative activity of free *Gc*APRD in the presence of Co^{2+} and Ni^{2+} 3

Fig. S1 Relative activity of free *Gc*APRD in the presence of Co^{2+} and Ni^{2+} 3

3. Elution of *Gc*APRD nanocrystal by imidazole....... 4

Fig. S2 Elution of *Gc*APRD nanocrystal by 500 mM imidazole 4

4. Deactivation of *Gc*APRD in the *Gc*APRD nanocrystal 4

5. *Gc*APRD nanocrystal characterization by EDX analysis....... 4

Fig. S3 SEM image and EDX analysis of *Gc*APRD nanocrystal....... 5

Fig. S4 SEM image and EDX analysis of $Co_3(PO_4)_2$ crystal (control) 6

6. *Gc*APRD nanocrystal characterization by TGA....... 7

Fig. S5 TGA curve of *Gc*APRD nanocrystal....... 7

7. ^{1}H NMR spectrum of (*S*)-6b....... 8

Reference 9

1. Experimental section

Table S1 Conditions of *Gc*APRD nanocrystal preparation

Figure	Metal ions concentration (mM)	PBS concentration (mM)	PBS pH	Protein concentration (mg/mL)	Specific activity of free *Gc*APRD (μmol/min/μg)
**1a**	10	3.75	7.4	1	25.7 ± 2.5
**1b**	-	3.75	7.4	1	33.4 ± 0.0
**1c**	10	-	7.4	3	40.2 ± 2.2
**1d**	10	3.75	-	1	23.7 ± 2.6
**1e**	10	3.75	9.2	-	23.7 ± 2.6

Table S2 Chiral GC retention time of ketone, alcohol, and ester standards

Compound	Conditions	Retention time (min)				
		Ketone (**a**)	Alcohol (**b**)		Propionate (**c**)	
			S	*R*	*S*	*R*
**1a**	a	10.0	14.9	14.5	-	-
**2a**	b	15.2	18.6[1]		20.5	21.1
**3a**	c	7.7	16.6[1]		19.0	19.6
**4a**	b	14.6	18.6[1]		20.1	20.6
**5a**	d	9.8	12.4	12.1	-	-
**6a**	e	14.8	21.9	20.9	-	-
**7a**	f	17.7	23.3[1]		26.0	26.9

a: 40 ℃ 1 min 1 ℃/min 120 ℃ 10 min (internal standard retention time 6.5 min)

b: 40 ℃ 5 min 5 ℃/min 120 ℃ 5 min (internal standard retention time 12.3 min)

c: 40 ℃ 10 min 5 ℃/min 120 ℃ 5 min (internal standard retention time 16.4 min)

d: 40 ℃ 1 min 10 ℃/min 150 ℃ 10 min (internal standard retention time 6.4 min)

e: 40 ℃ 1 min 10 ℃/min 160 ℃ 10 min (internal standard retention time 6.4 min)

f: 40 ℃ 1 min 10 ℃/min 140 ℃ 10 min (internal standard retention time 6.3 min)

[1] *S* and *R* peaks were not separated well, thus propionylation reaction was conducted.

2. Relative activity of free *Gc*APRD in the presence of Co^{2+} and Ni^{2+}

Relative activities of free *Gc*APRD in the presence of 10 mM Co^{2+} and Ni^{2+} are shown in **Figure S1**. It was found that the presence of Ni^{2+} caused the decrease in *Gc*APRD relative activity to 43%, while the presence of Co^{2+} retained 95% relative activity. This result strongly suggests that the presence of 10 mM Ni^{2+} during the nanocrystal formation process deactivated *Gc*APRD, resulted in poor activity of *Gc*APRD nanocrystal formed by Ni^{2+}.

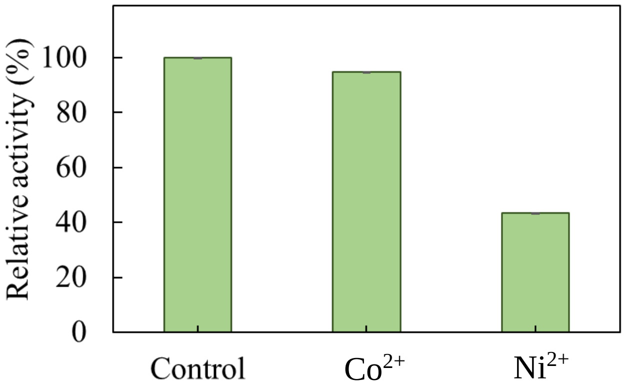

Fig. S1 Relative activity of free *Gc*APRD in the presence of Co^{2+} and Ni^{2+}. *Gc*APRD (0.27 mg/mL protein) was incubated with the presence of Co^{2+} and Ni^{2+} (10 mM) at 4 ˚C for 16 h. The relative activity measurement conditions are described in section **2.4**, by using **1a** (3.0 mM) and free *Gc*APRD (0.27 µg of protein/mL). The specific activity of control was determined to be 41.04 µmol/min/µg.

3. Elution of *Gc*APRD nanocrystal by imidazole

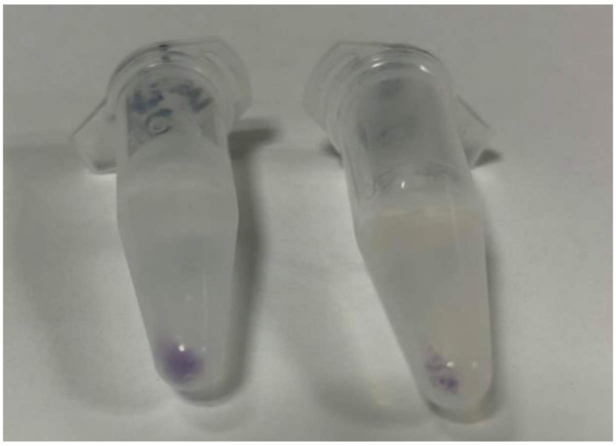

Fig. S2 Elution of *Gc*APRD nanocrystal by 500 mM imidazole.
Left: Non-eluted *Gc*APRD nanocrystal,
right: eluted *Gc*APRD nanocrystal.

4. Deactivation of *Gc*APRD in the *Gc*APRD nanocrystal

Role of *Gc*APRD in the *Gc*APRD nanocrystal was clarified by the following experiment. The *Gc*APRD in the *Gc*APRD nanocrystal was deactivated by incubating the *Gc*APRD nanocrystal at 100 ˚C for 30 min, and the activity was measured. It was found that *Gc*APRD nanocrystal lost its activity completely after enzyme deactivation, which strongly proved that the presence of active *Gc*APRD was mandatory for the *Gc*APRD nanocrystal activity.

5. *Gc*APRD nanocrystal characterization by EDX analysis

The EDX analysis of *Gc*APRD nanocrystal (**Figure S3**) and $Co_3(PO_4)_2$ crystal (control) (**Figure S4**) were performed. **Figure S3b-g** and **Figure S4b-g** presents the elemental mapping of *Gc*APRD nanocrystal and $Co_3(PO_4)_2$ crystal without *Gc*APRD, consist of carbon, oxygen, nitrogen, cobalt and phosphorous, respectively. **Figure S3h** shows the elemental analysis of *Gc*APRD nanocrystal by EDX. It was found that the *Gc*APRD nanocrystal consists of carbon as the highest atomic percentage at 42%, while the $Co_3(PO_4)_2$ crystal only presents 13% atomic percentage of carbon (**Figure S4h**). The higher atomic percentage of carbon in the *Gc*APRD

nanocrystal compared with $Co_3(PO_4)_2$ crystal suggests the co-localization of *Gc*APRD and $Co_3(PO_4)_2$. The presence of carbon and nitrogen in the $Co_3(PO_4)_2$ crystal elemental mapping was possibly from the added HEPES buffer with the absence of enzyme during the formation process.

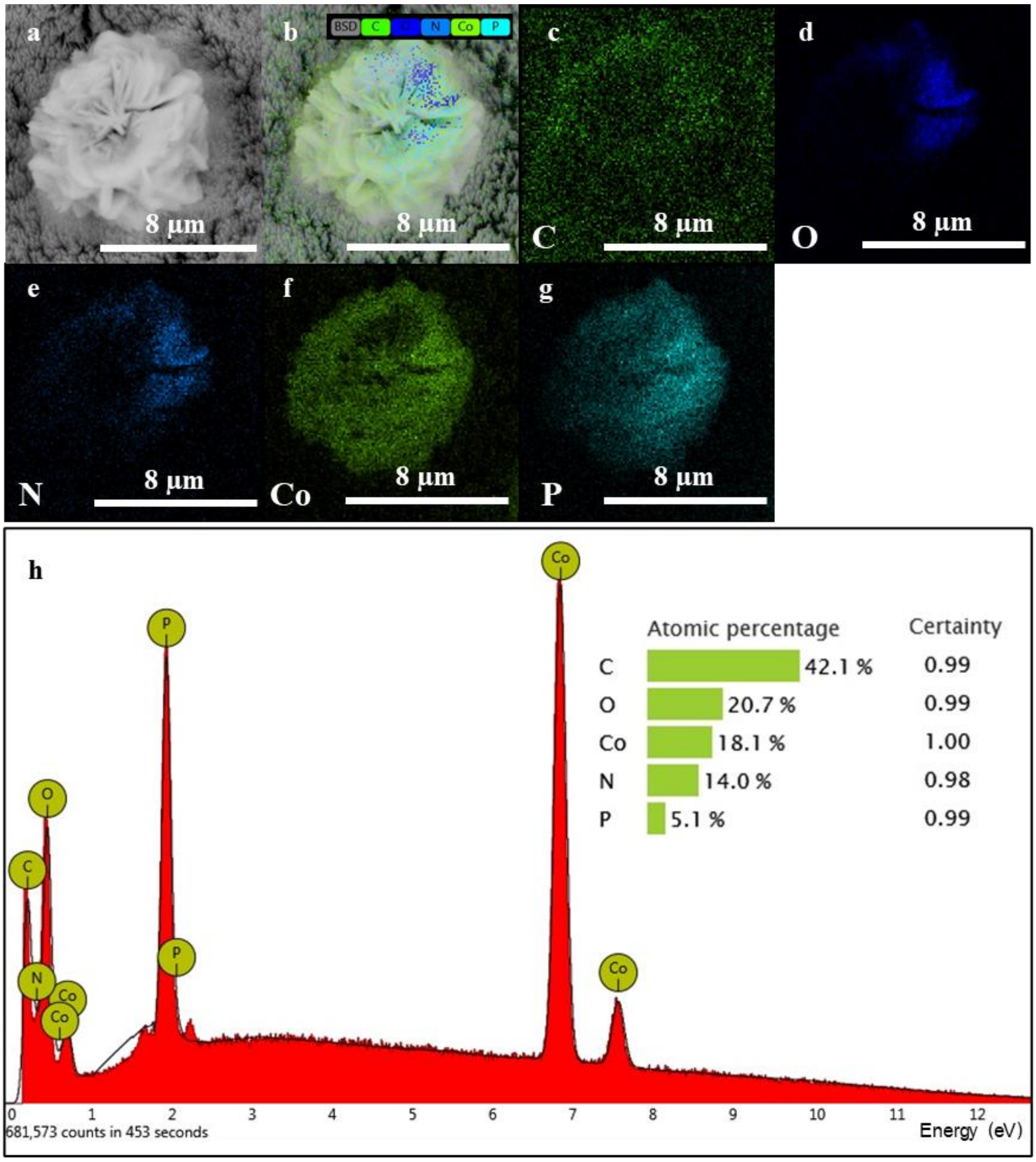

Fig. S3 SEM image and EDX analysis of *Gc*APRD nanocrystal. a) SEM image of *Gc*APRD nanocrystal, b) elemental mapping image of *Gc*APRD nanocrystal, c) carbon mapping, d) oxygen mapping, e) nitrogen mapping, f) cobalt mapping, g) phosphorous mapping, and h) elemental analysis of *Gc*APRD nanocrystal by EDX.

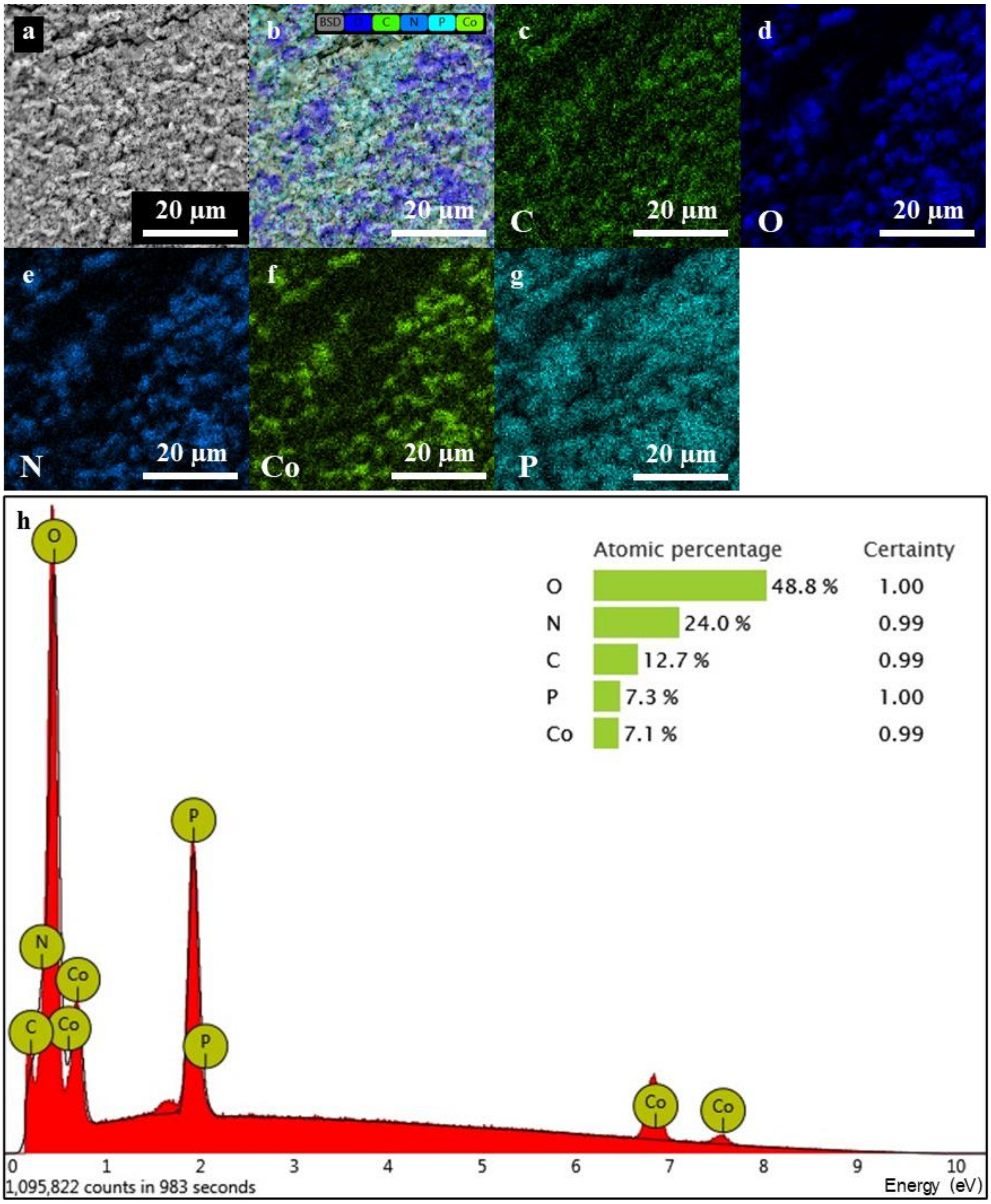

Fig. S4 SEM image and EDX analysis of $Co_3(PO_4)_2$ crystal (control). a) SEM image of $Co_3(PO_4)_2$ crystal, b) elemental mapping image of $Co_3(PO_4)_2$ crystal, c) carbon mapping, d) oxygen mapping, e) nitrogen mapping, f) cobalt mapping, g) phosphorous mapping, and h) elemental analysis of $Co_3(PO_4)_2$ crystal by EDX.

6. *Gc*APRD nanocrystal characterization by TGA

TGA curve of *Gc*APRD nanocrystal is demonstrated in **Figure S5**. The weight loss of 10.14% between 40 ˚C and 250.51 ˚C corresponds to the loss of free water and bound water of the *Gc*APRD nanocrystal. The weight loss between 250.51 ˚C and 572.85 ˚C, corresponds to the pyrolytic decomposition of *Gc*APRD. This TGA curve supports the presence of the *Gc*APRD in the nanocrystal.

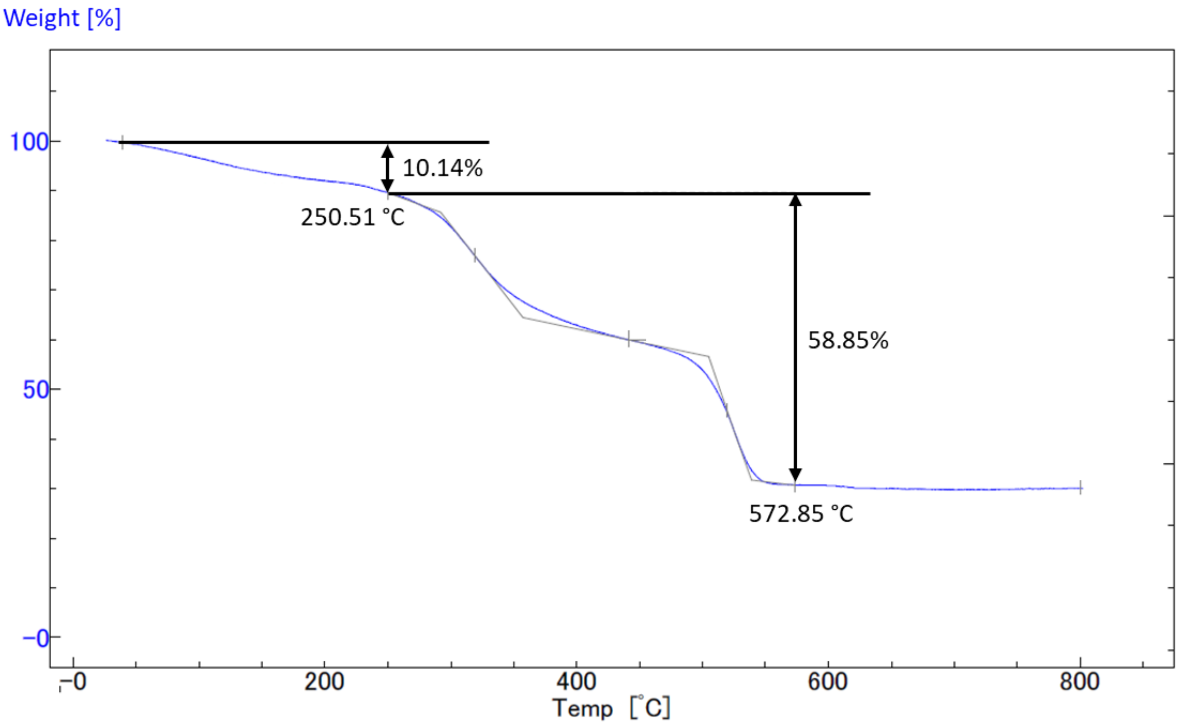

Fig. S5 TGA curve of *Gc*APRD nanocrystal. The experiment was performed, with the method described in literature,[1] by increasing temperature from 40 ˚C to 800 ˚C with a heating rate of 10 ˚C/min under air atmosphere.

7. ^{1}H NMR spectrum of (*S*)-6b

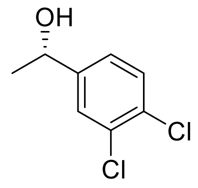

(*S*)-6b

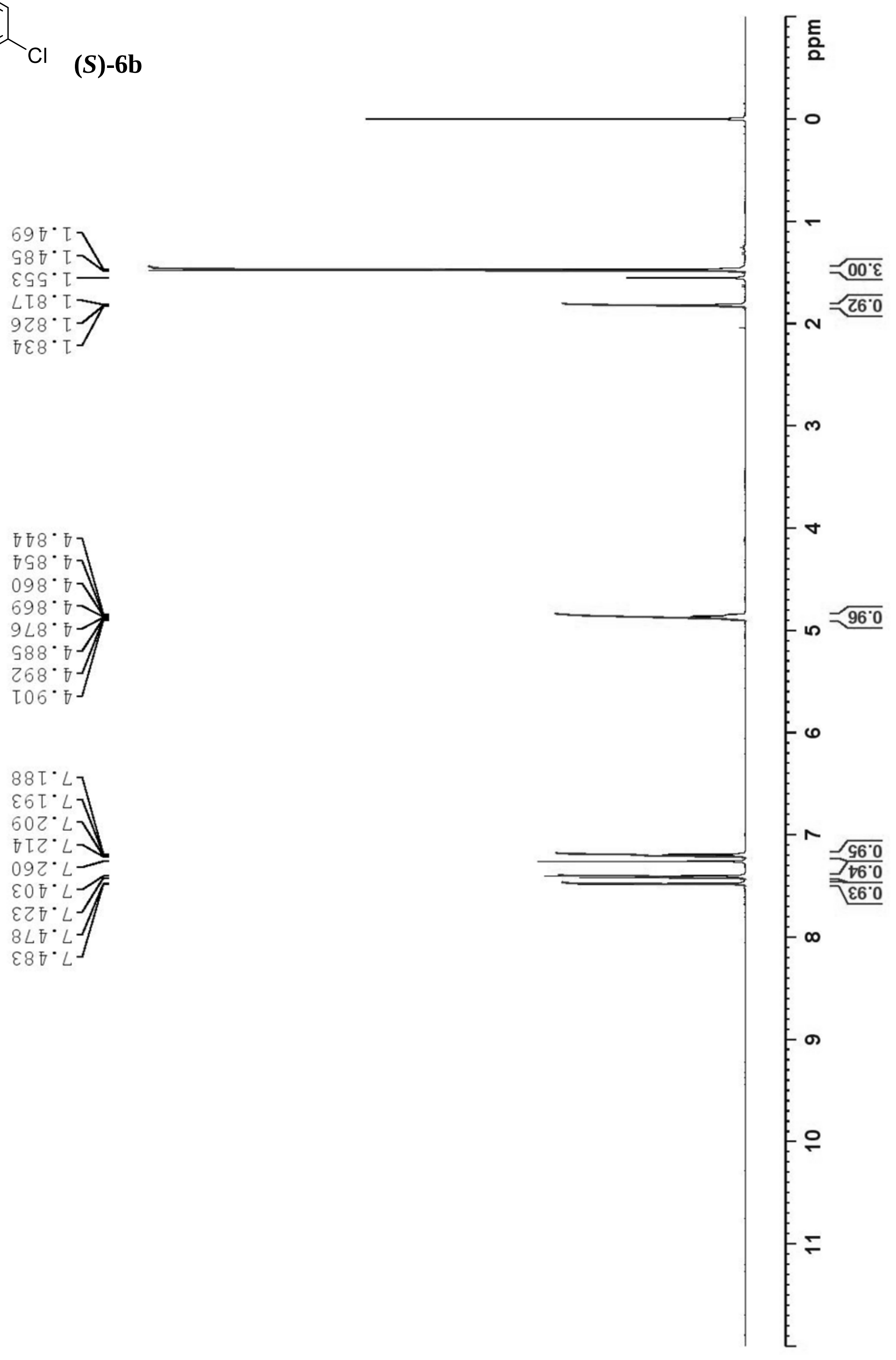

Reference

1 Y. Zhang, W. Sun, N. M. Elfeky, Y. Wang, D. Zhao, H. Zhou, J. Wang and Y. Bao, *Enzyme Microb. Technol.*, 2020, **132**, 109408.
